# Spontaneous Rupture and Hemorrhage of WON: A Case Report

**DOI:** 10.3389/fsurg.2022.906520

**Published:** 2022-06-23

**Authors:** Jican Yan, Wenhao Yu, Jingxin Yan, Xinjian Guo, Lizhao Hou, Li Ren, Haining Fan

**Affiliations:** ^1^ Department of Hepatopancreatobiliary Surgery, Qinghai University Affiliated Hospital, Xining, China; ^2^ Department of Postgraduate, Qinghai University, Xining, China; ^3^ Department of Interventional Therapy, Qinghai University, Xining, China; ^4^ Department of Pathology, Affiliated Hospital of Qinghai University, Xining, China; ^5^ Department of Hepatopancreatobiliary Surgery, Qinghai University Affiliated Hospital, Qinghai Key Laboratory of Hydatid Research, Xining, China

**Keywords:** WON, spontaneous rupture, hematoma, surgical treatment, case report

## Abstract

**Background:**

Pancreatic pseudocysts are characterized by the leakage of pancreatic juice caused by various reasons, which leads to pancreatic juice accumulates around the pancreas, and stimulates the greater omentum and other tissues to form an area of fibrotic loculated effusion with an integrated capsule. Approximately, one-third of patients experienced recurrent pancreatic juice leakage, compression symptoms, infection, and bleeding, which requires surgical intervention, but spontaneous rupture cases are extremely rare.

**Case presentation:**

We here present the case of 40-year-old male who presented with abdominal pain and vomiting for two days and 10 h, respectively. He had a history of chronic pancreatitis and pseudocysts. The symptoms of abdominal pain worsened in the second day. Laboratory tests showed a progressive decrease in hemoglobin. Then, emergency pancreatoduodenectomy was performed. Intraoperative exploration found a small blood clot in the abdominal cavity and a hematoma that had formed in the intestinal cavity and retroperitoneum.

**Conclusion:**

This case showed that pseudocysts of the pancreas can rupture under certain circumstances, leading to intraperitoneal bleeding and hematoma formation, which can endanger the life of the patient. And surgical treatment can be the first choice for hemorrhagic pseudocysts.

## Introduction

Pancreatic pseudocyst (PPC) is a common local complication of pancreatitis. Acute pancreatitis, chronic pancreatitis, traumas, and pancreatic surgery may lead to leakage of pancreatic juice and pancreatic juice accumulates around the pancreas, stimulating the greater omentum and other tissues to form a fibrotic mass with a complete capsule but no epithelial tissue, which is called a pseudocyst (1, 2). Pancreatic pseudocyst can be a delayed (usually >4 weeks) complication of interstitial oedematous pancreatitis and necrosis, which may lead to an acute necrotic collection (ANC) or/and walled-off necrosis (WON). ANC contains amounts of leakage of pancreatic juice and necrosis associated with necrotizing pancreatitis; the necrosis can spread to pancreatic parenchyma and/or the peripancreatic tissues. After the tunica is completely walled, it is called WON. WON usually occurs after onset of necrotizing pancreatitis(usually >4 weeks) (1). Pseudocyst formation usually takes more than 2 weeks after the onset of the disease, and it takes 4–6 weeks for the cyst wall to mature (1, 2). It has been reported in literature that pancreatic pseudocysts have a certain possibility of spontaneous regression, and the probability of regression within 6 weeks is greater than 50%; even if it persists for more than 6 weeks, approximately 8% of pancreatic pseudocysts can spontaneously regress (2). Approximately 1% to 4% of patients with pseudocysts of the pancreas may develop an intracapsular infection, at which time fever and other complication may occur. Some cysts may break into the stomach, duodenum, chest or/and anterior abdominal wall to form intrabdominal and external pancreatic fistulas.

Spontaneous rupture and hemorrhage of WONs and hematoma formation in the intestinal cavity and retroperitoneum are extremely rare. We herei report a case of a 40-year-old man who was considered to have an unusual case of WON with retroperitoneal hematoma formation. Pancreaticoduodenectomy was performed in an emergency and providing data regarding the treatment and diagnosis.

## Case Presentation

**Figure 1:**
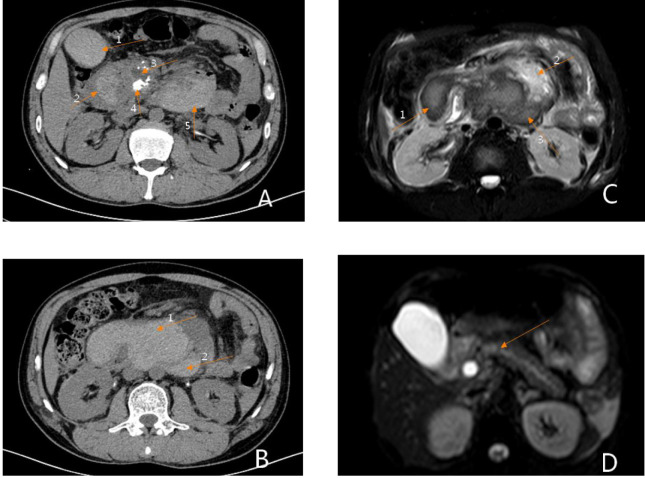
Preoperative imaging findings. (**A**) Phase III dynamic enhanced abdominal CT: Arrow 1 shows an intragallbladder hematoma, arrow 2 shows an intraduodenal hematoma, arrow 3 shows dilation of the main pancreatic duct, arrow 4 shows uneven enhancement of the head of the pancreas, suggestive of pancreatitis, and arrow 5 shows a retroperitoneal hematoma. (**B**) Phase III dynamic contrast-enhanced abdominal CT: Arrow 1 shows an intraintestinal hematoma, arrow 2 shows a retroperitoneal hematoma. (**C**) Abdominal MRI: Arrow 1 shows an intraduodenal hematoma, arrow 2 shows normal pancreatic tissue, and arrow 3 shows a retroperitoneal hematoma. (**D**) Abdominal MRI: This image shows a fully distended pancreatic duct.

The 40-year-old male was presented to the hospital on December 9, 2020 with the chief complaint of “upper abdominal pain accompanied with vomiting for 2 days and aggravation for 10 h”. The abdomen was flat, with positive tenderness in the upper abdomen, accompanied by rebound pain. Grey-Turner or Cullen sign was negative.The laboratory results were as follows: White blood cell count (WBC): 15.43*10*9/L, hemoglobin (HGB): 113 g/L, AMY (Amylase) 398 U/L, lipase (LIP) 626.0 U/L, Serum calcium ion (Ca): 2.36 mmol/L, Glucose (Glu): 7.6 µmol/L, Lactate-dehydrogenase(LDH): 177U/L, C-reactive-protein(CRP): 141 mg/L, Prothrombin-time(PT): 11.2 s, Activated-partial-thromboplastin-time(APTT): 36.3 s. On the second day after admission, the patient complained of increased abdominal pain, and emergency blood tests showed HGB 75 g/L. The second day after hospitalization, the patient complained a more violent abdominal pain, and the enhanced abdominal CT examination showed the formation of a hematoma in the segment at the duodenal level with the range of 13.28*7.52 cm, and CT value was about 66 Hu. The hematoma located in the duodenal intestinal canal, so the possibility of intestinal rupture was considered (Figures 1A,B). Dynamic hepatic MRI examination showed the formation of a very large retroperitoneal hematoma (acute-subacute stage), with adhesions to the pancreatic head and duodenum, unclear decomposability, and hematoceles in the bilateral paracolic sulcus (Figures 1C,D).

**Figure 2:**
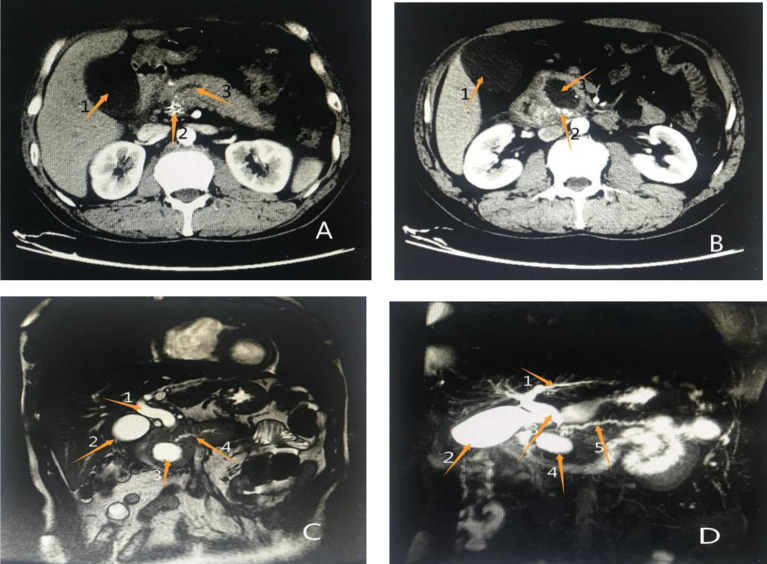
Results of previous imaging examinations at another hospital (**A**) (enhanced abdominal CT): Arrow 1 shows an enlarged gallbladder, arrow 2 shows patchy enhancement of the pancreatic head, and arrow 3 shows a significantly dilated main pancreatic duct. (**B**) Enhanced abdominal CT: Arrow 1 shows an enlarged gallbladder, arrow 2 shows patchy enhancement of the pancreatic head, suggestive of pancreatitis, and arrow 3 shows a rounded, low-density shadow of the pancreatic head, considered a pseudocyst. (**C**) Abdominal MRI: Arrow 1 shows a dilated common bile duct, arrow 2 shows an enlarged gallbladder, arrow 3 shows a pseudocyst of the pancreatic head, and arrow 4 shows full dilation of the main pancreatic duct. (**D**) Abdominal MRCP: Arrow 1 shows a dilated intrahepatic bile duct, arrow 2 shows an enlarged gallbladder, arrow 3 shows a dilated common bile duct, arrow 4 shows a pseudocyst of the pancreatic head, and arrow 5 shows a fully dilated main pancreaticduct.

The patient reported a previous history of chronic pancreatitis, which is considered alcoholic pancreatitis. He had a history of smoking and alcohol abuse for more than 20 years. He smoked approximately 10 cigarettes per day. He quitted smoking and alcohol for more than 5 years. Two years ago, the patient had undergone enhanced abdominal CT and abdominal MRI in the local hospital. which showed: 1.dilatation of bile ducts inside and outside the liver with 18 mm in diameter of common bile duct; 2. enlargement of gallbladder; 3. dilatation of main pancreatic duct and enlargement of pancreatic head; 4. patchy dense shadow and round low-density shadow in the inside with a max diameter of 39 mm and no enhancement in low-density. The abdominal MRI examination showed: dilatation of common bile duct, engorgement of pancreas, high signal shadow in DWI sequence, irregular T1 and T2 signal shadow in the head of pancreas, long T1 and long T2 signal shadow in the inside, the range of about 55*45*46 mm, enhanced scan showed obvious enhancement, the main pancreatic duct dilated. A diagnosis of chronic pancreatitis with WON, dilatation of intrahepatic and extrahepatic bile ducts, and dilatation of the main pancreatic duct (Figures 2A–D) was made.

**Figure 3:**
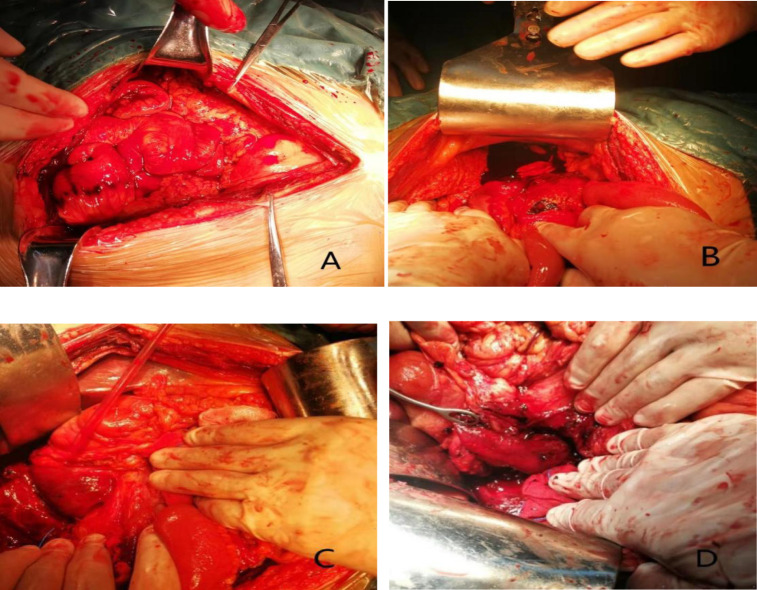
Intraoperative images. (**A, B**) A small amount of uncoagulated blood observed in the abdominal cavity after creating an incision in the peritoneum incision. (**C**) Hematocele below the hepatic margin observed during intraoperative exploration. (**D**) A dark blood clot in the posterior peritoneum of the duodenum.

**Figure 4:**
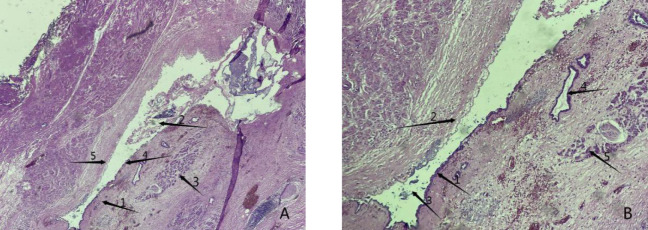
Postoperative histopathology results. (**A**) Arrow 1 shows the capsule of the cyst wall, arrow 2 indicates staining for the secretory protein from the cystic cavity and pancreatic juice, indicating organization, arrow 3 indicates a normal pancreatic gland structure, and arrow 4 indicates a normal residual epithelioid structure in the process of epithelial shedding (40× magnification). (**B**) Arrow 1 shows a normal residual epithelioid structure, arrow 2 shows epithelial detachment of the pancreas, arrow 3 shows the cystic cavity and its secreted proteins, pancreatic fluid and other tissues, arrow 4 shows a normal pancreatic duct structure, and arrow 5 shows a normal pancreatic gland structure (100× magnification).

**Figure 5:**
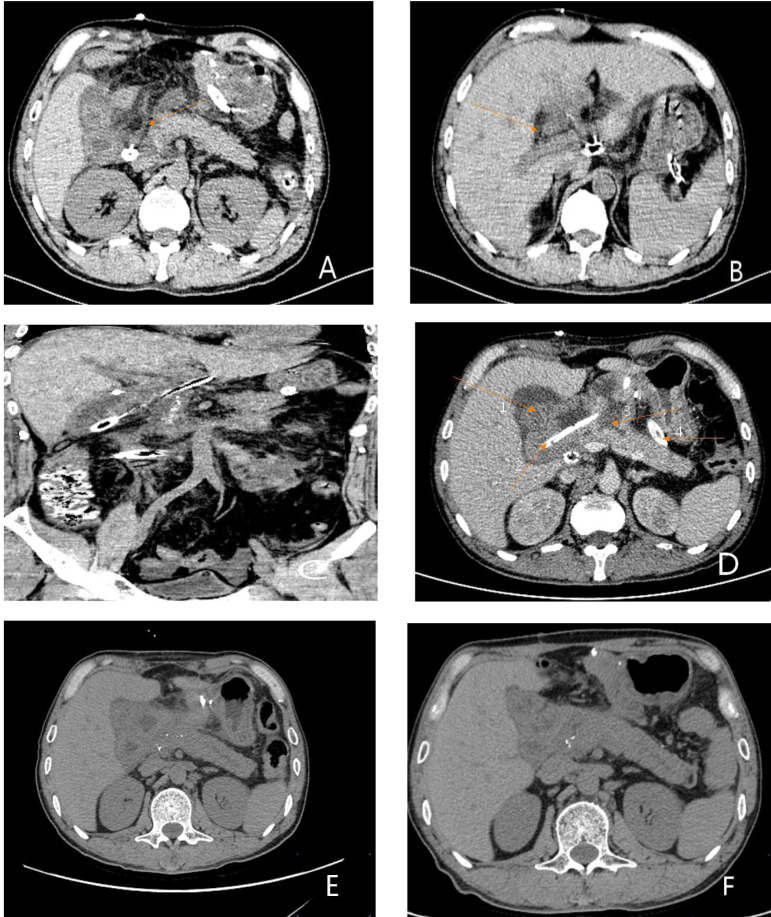
Postoperative CT results. (**A**) Plain CT scan of the whole abdomen on the 3rd postoperative day. The arrow shows the pancreaticojejunostomy. (**B**) Full abdominal CT reexamination on day 3 postoperatively. The arrow showed the pancreaticojejunostomy. (**C**) Full abdominal CT reexamination on day postoperatively showing the entire pancreaticoduodenectomy area. (**D**) Plain CT scan of the whole abdomen on day 11 postoperatively. Arrow 1 shows the biliary and intestinal anastomosis, arrow 2 shows the indwelling open-field decompression tube intraoperatively, arrow 3 shows the pancreaticojejunostomy, and arrow 4 shows the indwelling three-cavity irrigation and drainage tube intraoperatively. (**E**) Plain CT scan of the whole abdomen 1 month after surgery showing no obvious abnormalities. (**F**) Plain CT scan of the whole abdomen 3 months after surgery indicating good recovery.

Combined with the patient’s history and the results of related auxiliary examinations, the patient was diagnosed with acute severe pancreatitis, a very large retroperitoneal hematoma, WON, pancreatic duct dilatation and stones, common bile duct dilatation, and chronic cholecystitis. The patient met the indications for exploratory laparotomy and emergency surgery, and pancreaticoduodenectomy was planned. Emergency surgery was performed at 20:50 on December 11, 2020. Intraoperative exploration revealed intraabdominal bleeding (Figures 3A,B), serious abdominal adhesions, and a hematoma with a size and location in agreement with those observed on preoperative imaging. In the process of separating the adhesions after observing the very large retroperitoneal hematoma (Figure 3D) and removing a large number of blood clots, the outline of the pancreas became clear, and pancreatic duct expansion, with stones in the main pancreatic duct, and visible peripancreatic leakage were observed, as well as a pseudocyst of the head of pancreas that was closely related to the hematoma. In view of the above situation, the patient was treated with pancreaticoduodenectomy, retroperitoneal hematoma removal, abdominal irrigation and drainage, and reconstruction of the digestive tract. The patient underwent ECG monitoring, oxygen supplementation, protective liver treatment, nutritional support, blood volume replenishment, water, electrolyte and acid-base balancing and other symptomatic supportive treatment. About 1,500 mL of blood was lost during the operation, and 800 mL of suspended red blood cells and 850 mL of plasma were transfused during the operation. Postoperative frozen pathology indicated atrophy of pancreatic glands, cord-like and slab-like proliferation of pancreatic islet cells, a small amount of residual epithelium in the cyst wall, hyperemia and edema under the pancreatic capsule, and infiltration of a large number of neutrophils, which are consistent with the diagnosis (Figures 4A,B). On the 3rd day after surgery, plain CT of the abdomen and pelvis showed multiple areas of exudation in the pelvic, a small protohematoma, and increased area of effusion around the liver and bilateral paracolic sulcus (Figures 5A,B). Reexamination of the abdomen and pelvic cavity by CT 11 days after surgery showed multiple areas of exudation around the duodenum compared with the previous scan, and the abdominal hematoma was smaller and less dense than before (Figure 5D). The patient was discharged from the hospital on the 15th day after surgery. Upon discharge, the patient was instructed to undergo follow-up examinations of liver and kidney function, as well as abdominal CT and other related tests and examinations regularly, and to pay attention to disease progression. The results of regular follow-up and relevant examinations showed that the patient recovered well after surgery (Figures 5E,F), with a normal sleep and defecation.

## Discussion

In our opinion, the main concerns in this case are the risk factors of pseudocyst rupture and the choice of the surgical method. Lesion rupture is one potential complication of cystic pancreatic disease. Pujitha Kudaravalli et al pointed out that the mortality of pancreatic hemorrhagic pseudocysts is around 40%, and approximately 13% of pancreatic pseudocysts may be associated with hemorrhage (3). The mechanism of rupture may be continuous pancreatic necrosis and exudation, resulting in a continuous increase in pathological contents, excessive internal pressure, overload, and thus rupture. Alternatively, the cyst itself or the cystic contents erode the blood vessels, causing blood vessel rupture and bleeding.Besides, portal hypertension could be caused by cystic compression or vascular embolism, leading to varicose vein ruptured and bled. In addition, if the abdomen is attacked by external forces, cystic lesions may rupture (4, 5).

Currently, there are many treatment options for pancreatic pseudocysts, such as conventional conservative treatment, endoscopic treatment, percutaneous catheter drainage and surgical treatment. According to the relevant literature and guidelines, conservative treatment is appropriate for patients with no obvious symptoms, no complications and no trend of pancreatic pseudocyst growth; in general, cysts <6 cm can be absorbed, so in such patients with PPC but no obvious signs or symptoms. In treatment of these patients, extended observation with regular follow-ups to review the cyst size may be appropriate (6). Percutaneous puncture drainage is suitable for large-diameter cysts or cysts that grow rapidly to relieve persistent pain, especially in high-risk patients who cannot tolerate surgery; however, because of the high change of relapse, infection, hemorrhage, perforation, pancreatic fistula and various complications (7, 8), this procedure has become less common in recent years. It has been reported that the recurrence rate of pancreatic pseudocysts treated with percutaneous catheter drainage is about 30% (8). With the development of endoscopic technology, endoscopic ultrasound-guided puncture and drainage has gradually become the main method for the treatment of pancreatic pseudocysts. Endoscopic drainage has the advantages of low complication and recurrence rates, a good treatment effect, minimal trauma, a short hospitalization time and low treatment costs, which is mainly suitable for patients with pseudocyst wall maturation and a cyst formation time of >6 weeks or a cyst diameter of >5 cm. Studies have reported that the success rate of endoscopic ultrasound-guided puncture and drainage for the treatment of PPCs is 89.3%, with no recurrence in any patient (9, 10). Surgical treatment should be actively performed in patients with conservative treatment failure, aggravated signs of peritonitis, endoscopic, and/or interventional therapy failure. Intractable abdominal pain is the main surgical indication (11). With the continuous development of laparoscopic techniques, laparoscopic pseudocyst–digestive tract anastomosis has gradually become accepted by surgeons and patients. Some scholars have also confirmed the advantages of laparoscopic surgery compared with open surgery (12). The laparoscopic treatment of PPCs is suitable for patients with mature cyst walls, complications and PPCs that cannot be absorbed, as well as patients in good health. The main surgical methods include pancreatectomy, cyst-gastric anastomosis, cyst-duodenal anastomosis, cyst-jejunal anastomosis, and the specific surgical method applied should be determined up to the location and orientation of the pseudocyst. Cyst jejunostomy is the commonly used in clinical practice, has the best drainage effect and the fewest postoperative complications, which is an ideal surgical method for the treatment of PPCs; this is more suitable for patients with cysts that have formed thick walls, especially those with cysts located far from the gastroduodenum and are not suitable for resection (11–13). It has been reported that complications such as abdominal infection, inadequate anastomosis and gastric perforation may occur during laparoscopic surgery, which limits the clinical application of laparoscopic surgery to a certain extent (12).

Currently, the methods used in the surgical treatment of chronic pancreatitis include pancreatoduodenectomy (PD)/pylorous-preserving pancreaticoduodenectomy (PPPD) and duodenum-preserving pancreatic head resection (DPPHR). Professor Beger first proposed duodenum-sparing pancreatectomy in 1972 (14), which was initially applied in the treatment of chronic pancreatitis and later gradually applied in the treatment of benign and low-grade malignant neoplastic lesions of the head of the pancreas with the derivation of different improved surgical methods. Compared with PD/PPPD, DPPHR retains the standard of the gastroduodenum and biliary tract and has certain advantages in improving postoperative quality of life. At present, the clinical application of DPPHR is limited. It has been pointed out that such benign and low-grade malignant pancreatic diseases for which DPPHR is applicable have a high risk of canceration. Even if the lesions are surgically removed, there is still a possibility of postoperative canceration, and further surgical intervention is needed if canceration occurs. PD is the most effective treatment for malignant tumors of the head of the pancreas (11, 12, 14). Besides, DPPHR is a difficult operation to perform and requires a high level of surgical skill. The most important aspect is preservation of the duodenum; the difficulty of preserving the duodenum lies in ensuring that the blood supply of the duodenum is not damaged, and attention should be given to protecting the blood supply of the bile duct to avoid bile leakage caused by a poor blood supply.

In summary, there are various treatment methods for pancreatic cystic lesions, and the patient’s condition and actual situation should be flexibly mastered to achieve individualized treatment when making treatment plans. The author believed that the patient had continued pancreatic necrosis and exudation, with increasing pathological contents, high internal pressure, chyme of vascular wall, blood vessel rupture, spontaneous rupture and hemorrhage, and hematoma formation in the intestinal cavity and retroperitoneum. Emergency pancreaticoduodenectomy can not only stop bleeding in time but also effectively solve the problem of severe infection. It is undoubtedly an effective treatment for patients with spontaneous rupture and hemorrhage of WON.

## Data Availability

The original contributions presented in the study are included in the article/Supplementary Material, further inquiries can be directed to the corresponding author/s.
